# Can mobile genetic elements rescue genes from extinction?

**DOI:** 10.1007/s00294-020-01104-9

**Published:** 2020-09-03

**Authors:** Bram van Dijk

**Affiliations:** Max Planck Institute for Evolutionary Biology, Plön, Germany

**Keywords:** Horizontal gene transfer (HGT), Mobile genetic elements (MGEs), Selfish genetic elements (SGEs), Evolution, Bacterial fitness, Slightly beneficial genes

## Abstract

Bacteria and other prokaryotes evolve primarily through rapid changes in their gene content by quickly losing and gaining genes whenever an ecological opportunity emerges. As gene loss and horizontal gene transfer (HGT) appear to be the most common events across the prokaryotic tree of life, we need to think beyond gradual sequence evolution if we wish to understand the microbial world. Especially genes that reside on mobile genetic elements (MGEs) may spread much more rapidly through a microbial population than genes that reside on the bacterial chromosome. This raises the question: why are some genes associated with MGEs, while others are not? Here, I briefly review a recently proposed class of genes for which we have coined the term “rescuable genes”. The fitness effect of carrying these genes is so small, either constantly or on average, that they are prone to be lost from a microbial population. I argue that HGT, even when costly to the individual cells, may play an important role in maintaining these rescuable genes in microbial communities.

Through processes collectively referred to as Horizontal Gene Transfer (HGT), bacteria and other microorganisms can acquire new genes from other individuals. Precisely how genes are transferred from one individual to the next, changes depending on the exact mechanism of HGT, yielding a diverse repertoire of mobile genetic elements (MGEs) in nature. Interestingly, MGEs appear to be on a continuum of parasitism and mutualism (Harrison et al. 2015), aiding the transfer of ecologically significant genes on the one end (Jain et al. 2003; Wiedenbeck and Cohan 2011; Quistad et al. 2020; Casacuberta and González 2013; Mell and Redfield 2014; Niehus et al. 2015; Lopatkin et al. 2016), and being costly or otherwise disruptive when they merely replicate for their own gain (Vogan and Higgs 2011; Baltrus 2013; Werren 2011; Doolittle and Sapienza 1980).

Understanding the selection pressures that mobilise genes, and whether the genes and/or the host benefits from this mobilisation, is crucial if we wish to predict the evolution of microbial communities. This challenge can be dissected into two questions: (1) which genes can we expect to have increased gene mobility (Rankin et al. 2011; Abedon and LeJeune 2007; Bergstrom et al. 2000; Iranzo et al. 2017; Hall et al. 2016), and (2) what is the impact of increased gene mobility on the host (Stevenson et al. 2017; Hall et al. 2017; Nogueira et al. 2009)? In answering these questions, it is first important to consider that the horizontal transfer of genetic elements is very often a side-effect of other processes (Redfield 2001), such as infection by bacteriophages or illegitimate recombination during natural competence. However, whether HGT happens as a side-effect or not, it is still interesting to consider what are the types of traits that would benefit bacteria when transferred (Rankin et al. 2011).

From a population genetics perspective, HGT is often compared to processes like sex and recombination. It is, however, important to consider that HGT can also enable the copying of genes from one individual to the next, potentially even changing an individual's genotype during its lifetime. By means of this process, sometimes referred to as “additive” HGT (Choi et al. 2012; Thomas and Nielsen 2005; Soucy et al. 2015), DNA sequences can become Darwinian entities in their own right, i.e. have their own reproduction and survival dynamics. Self-replicating DNA elements like insertion sequences or transposons are precisely such Darwinian entities. These self-replicating DNA sequences either need to confer a benefit to their host (Park et al. 2020), or need to copy themselves from one individual to the next via additive HGT (Werren 2011; Doolittle and Sapienza 1980). But when do the hosts benefit from the transfer of MGEs, in light of all the risks associated with them?

Our recent modelling work has illustrated that additive HGT, even when costly, can help to retain slightly beneficial genes in a bacterial population (van Dijk et al. 2020). Due to ongoing mutations, genes with very small fitness effects are prone to extinction even in infinitely large populations (Eigen 1971), despite their de facto benefit. Additive HGT can, however, rescue these genes from extinction, and therewith improve the growth rate of a bacterial population. A bacterial population without additive HGT would eventually lose these “rescuable genes”, and would, therefore, be slightly less fit (see Fig. 1).

**Fig. 1:**
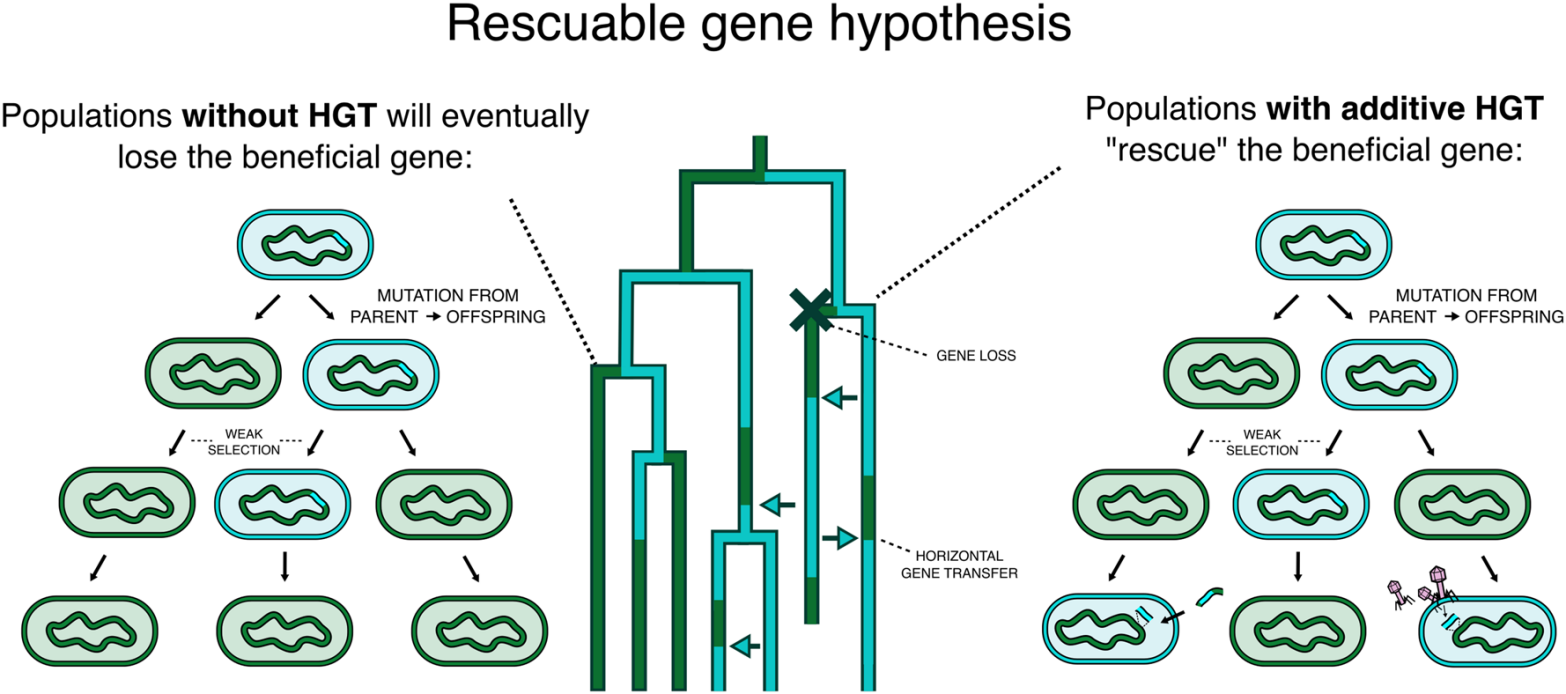
Cartoon visualisation of the rescuable gene hypothesis. Rescuable genes are genes that are so slightly beneficial that they would be lost in the absence of HGT. The rescuable gene hypothesis postulates that bacterial collectives can benefit from the additive spread of these genes through HGT (e.g. by encoding these genes on MGEs), even when this is costly for individual cells. As more beneficial genes are less dispensable, i.e. they are more readily maintained in the absence of HGT, spread through MGEs will only promote redundant gene copies

Intriguingly, our study also suggests that DNA uptake could be an evolved property of bacterial populations, as populations that engage in costly HGT would emerge from our computational model. Surprisingly, we found that individuals in spatially structured populations would even evolve DNA uptake in the presence of selfish genetic elements (SGEs), even when these SGEs were highly deleterious to the individual growth rate. This counter-intuitive result can be explained by the spatial structure in the model, as strains that are overwhelmed by deleterious SGEs will only locally go extinct while SGEs seek refuge in a new strain. In the meantime, other (non-infected) individuals will still experience selection pressures to maintain DNA uptake, as SGEs are not present in their current environment. The competition between spatially separated strains results in an interesting back-and-forth between highly competent cells that are at risk of SGE infection, and cells that do not engage in HGT but therefore lose their beneficial gene. In other words, a quasi-stable equilibrium is reached that can elegantly explain the coexistence of bacteria and SGEs.

It is of course still an open question if natural bacterial communities that engage in HGT do indeed retain more slightly beneficial genes than predominantly clonal communities. Our models, and the rescuable gene hypothesis that emerged from it, provides some interesting search images and testable hypotheses for experimental biology. For example, does phage predation or natural competence result in the predictable persistence of certain genes that would be lost in the absence of these processes? Are these genes indeed non-essential genes that are, on average, only slightly beneficial (van Dijk et al. 2020; Lehtinen et al. 2020)? Although very small fitness differences have been notoriously difficult to detect (Wiser and Lenski 2015; Bataillon 2000), new experimental techniques like DNA barcoding (Blundell and Levy 2014; Ba et al. 2019) and Hi-C metagenomics (Stevenson et al. 2017; Hall et al. 2017) may provide more data on which genes persist in complex microbial communities. By combining these exciting techniques with experiments that manipulate HGT in complex microbial communities (Quistad et al. 2020), we may soon know if HGT can indeed “rescue” genes from extinction.
